# Development of an Implementation Science Telehealth Toolkit to Promote Research Capacity in Evaluation of Telehealth Programs

**DOI:** 10.1089/tmr.2023.0039

**Published:** 2023-10-04

**Authors:** Emily E. Johnson, Ryan Kruis, Rebecca Verdin, Elana Wells, Dee W. Ford, Katherine R. Sterba

**Affiliations:** ^1^ College of Nursing, Medical University of South Carolina, Charleston, South Carolina, USA.; ^2^ Center for Telehealth, Medical University of South Carolina, Charleston, South Carolina, USA.; ^3^ Division of Pulmonary and Critical Care Medicine, Medical University of South Carolina, Charleston, South Carolina, USA.; ^4^ Department of Public Health Sciences, Medical University of South Carolina, Charleston, South Carolina, USA.

**Keywords:** telehealth, implementation science, dissemination, toolkit

## Abstract

**Background:**

The field of telehealth is rapidly growing and expanding access to quality health care, although there have been varied implementation outcomes in telehealth modalities. Dissemination and implementation (D&I) research can provide a systematic approach to identifying barriers and facilitators to telehealth implementation processes and outcomes.

**Methods:**

An interdisciplinary research and clinical team developed an implementation science telehealth toolkit to guide D&I evaluations of new and existing telehealth innovations.

**Results:**

The toolkit includes a separate section to correspond to each step in the D&I evaluation process. Each section includes resources to guide evaluation steps, telehealth specific considerations, and case study examples based on three completed telehealth evaluations.

**Discussion:**

The field of telehealth is forecasted to continue to expand, with potential to increase health care access to populations in need. This toolkit can help guide health care stakeholders to develop and carry out evaluations to improve understanding of telehealth processes and outcomes to maximize implementation and sustainability of these valuable innovations.

## Background

In recent years, utilization of telehealth modalities for health care delivery has expanded, both due to the COVID-19 pandemic and documented literature of the efficacy, acceptability, and value in these innovations.^1–7^ Despite the rapid expansion of telehealth, implementation has varied across care settings and specialties and among different patient populations, raising questions about equity and the resources required for quality telehealth programs.^8–11^ Thus, it is vital to understand implementation processes, characterize determinants of successful telehealth implementation, and define strategies for quality telehealth care across settings.

The field of dissemination and implementation (D&I) research has been showcased as key to accelerating the dissemination, implementation, and sustainability of evidence-based interventions into health care practice.^12,13^ D&I research is typically guided by frameworks, theories, and models with interdisciplinary research, clinical, and administrative teams using multiple methods to describe a variety of implementation processes and outcomes related to health care innovations. The multitude of approaches to D&I research can pose barriers to engagement in this field, but educational resources such as toolkits have been leveraged to increase engagement.^14^ The Agency for Healthcare Research and Quality (AHRQ) defines a “toolkit” as a collection of resources that can guide users in implementation of evidence-based practices or development of plans to reach evidence-based recommendations.^15^

While there are numerous existing D&I toolkits in the literature to support D&I evaluations of evidence-based interventions to health care practice, no D&I toolkits specifically focus on telehealth interventions.^16^ The field of D&I research offers many benefits to telehealth including providing a systematic approach to understanding telehealth outcomes and processes, demonstrating the value of telehealth programs, identifying challenges and success in telehealth delivery, facilitating utilization of data to overcome barriers and challenges, and guiding implementation strategies to improve patient and system outcomes.

Due to recent rapid telehealth expansion and benefits of the D&I field to contribute to efficient and effective telehealth implementation processes and outcomes, a team within the Telehealth Center of Excellence (COE) created a D&I telehealth toolkit to guide interprofessional telehealth teams to design and conduct D&I telehealth evaluations. One of the goals of the COE is to develop resources for telehealth providers, staff, and researchers to improve quality of telehealth innovations. This toolkit seeks to share best practices in D&I research steps and utilizes three completed telehealth evaluations to demonstrate D&I methodologies for telehealth innovations.^17–23^ The telehealth toolkit is intended to be a dynamic adaptable resource that can be utilized in a variety of telehealth settings by a broad group of telehealth stakeholders.

The objective of this manuscript is to describe the telehealth toolkit development process, content included in the toolkit, and applications to telehealth D&I evaluation. The toolkit and other COE resources can be accessed publicly at no cost online *via the Telehealth Center of Excellence website.*

## Methods

The development of the telehealth toolkit originated with an interdisciplinary team review of literature related to the evolving resources and tools in the D&I field. This literature review included an inventory of existing published D&I toolkits and examination of key components of D&I evaluation including common study designs, existing frameworks and models, implementation outcomes, and measures to assess those outcomes. Our team then framed the conceptualization of the telehealth toolkit with a series of interdisciplinary team (physicians, health services researchers, nurses, telehealth research coordinators) discussions to outline the format and content, after which two researchers (E.E.J. and K.R.S.) developed the content for each toolkit section. Throughout the process of content development, we followed the AHRQ toolkit checklist for content and usability to ensure sufficient information was presented in a clear format.^15^ The process of toolkit development was considered exempt from the university IRB.

After the initial toolkit draft was completed, our internal interdisciplinary telehealth team (*N* = 5) provided comprehensive review and feedback, which was used to update the toolkit content and formatting. Next, we completed an external review to assess toolkit acceptability and usability by telehealth experts, including three reviewers knowledgeable in the telehealth field. Participants reviewed the toolkit and completed an electronic survey. The survey included three items to rate usefulness of the toolkit to different audiences (scale of 1–4), seven questions assessing usability of the toolkit (scale of 1–100) and two questions rating content and clarity for each section of the toolkit (scale of 1–4). Open-ended feedback about each toolkit section was also collected.

## Results

### Toolkit content

**FIG. 1.**
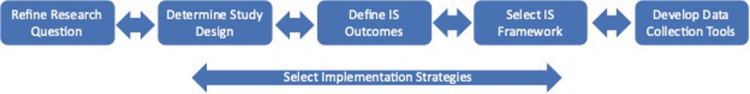
D&I telehealth toolkit sections; D&I evaluation process. D&I, dissemination and implementation.

Figure 1 illustrates the sections of the D&I telehealth toolkit and Table 1 describes the content within each section. Each toolkit section corresponds to a step in the D&I evaluation process: refine research question, determine study design, define implementation outcomes, select implementation framework, develop data collection tools and measures, and select implementation strategies. Each section includes consistent content to demonstrate the corresponding D&I evaluation step with introductory materials, practical questions for the research team, telehealth specific considerations, case study examples, and external articles and resources. The section on selecting implementation strategies is unique in that the steps in this section can be utilized throughout the D&I evaluation process to overcome identified barriers to implementation. Three case study examples from South Carolina telehealth programs were included throughout the toolkit, with one illustrative example in each section to further elucidate that specific D&I evaluation step.

**Table 1.** Sections of Telehealth Toolkit

Telehealth Toolkit content in each section	Description of content
Introductory Materials	Background information to define each D&I research step
Practical Questions to Guide Team	Key questions that a research team should consider during the current phase of research
Telehealth Specific Considerations	Relates the section content to the field of telehealth with examples
Case Study Examples	Provides a specific example of the D&I research step from one of three completed telehealth team projects to operationalize and illustrate the step
Key Articles and Resources	A reference list of external articles and websites to correspond with section materials

D&I, dissemination and implementation.

The first case study was an evaluation of a school-based telehealth asthma program. These types of telehealth programs have previously been demonstrated as cost-effective in improving asthma patient outcomes yet are challenging to implement.^24–26^ The D&I objective for this study was to characterize school-based telehealth asthma program delivery experiences and examine barriers and facilitators to telehealth program implementation.^20–23^

The second case study was an evaluation of a remote patient monitoring program to support diabetes management. While remote patient monitoring programs have exhibited success in improving patient diabetes outcomes, a program in South Carolina demonstrated variable implementation success across clinics.^27^ The objective for this D&I case study was to characterize clinic delivery strategies for a South Carolina remote patient monitoring program and to examine barriers and facilitators to program implementation in underserved and/or low-income community settings.^19^

The third case study was an evaluation of a program to treat perinatal and postpartum mental health and substance use disorders. These disorders are common across many populations, with high resulting morbidity and mortality.^28–31^ Although evidence-based treatment is available for these conditions, many barriers to care exist. Thus, the objective for this D&I study was to characterize barriers and facilitators to implementing a mental health and substance-use disorder telehealth program in community obstetric and pediatric clinics.^17,18^

### Internal and external review

The internal toolkit review guided changes to content, the order of sections, and overall formatting. Table 2 illustrates the feedback from the toolkit external review, which was also utilized to update the toolkit content and format. The toolkit content, clarity, and flow received high rankings from all three reviewers. In addition, the toolkit received high rankings in usability and recommendations for use by others. However, the ability of beginner D&I researchers to independently utilize the toolkit without insight from mentors or other resources received lower endorsement scores. These scores on usability for beginner researchers were considered acceptable as our team intends for this toolkit to serve as a supplemental guide to D&I telehealth evaluation and not replace expert mentorship.

**Table 2.** External Review of Telehealth Toolkit

Item	*Scale response set average (median, range); N = 3*	
Extent to which toolkit will be useful as a resource for	1–4 (1 = not very useful; 4 = extremely useful)	
D&I Beginners	3.7 (4, 3–4)	
D&I Intermediates	3.7 (4, 3–4)	
D&I Experts	2.7 (3, 2–3)	

Toolkit acceptability/usability ratings	1–100 (1 = completely disagree; 100 = completely agree)	
After reviewing the toolkit, I understand its content	90 (100, 70–100)	
This toolkit is easy to understand	88 (90, 78–95)	
The flow of the toolkit topics was appropriate	94 (100, 81–100)	
A person could use this toolkit and apply to the telehealth field without the help of a D&I expert	60 (60, 40–80)	
This toolkit provides all the resources/information that I need to know about D&I evaluations related to telehealth	73 (71, 57–90)	
I think this toolkit would be very useful for someone completing a D&I telehealth evaluation	93 (100, 78–100)	
I would recommend this toolkit to others who are going to carry out a D&I telehealth evaluation	97 (100, 90–100)	

Content and clarity ratings for toolkit sections	1–4 (1 = not very comprehensive/clear; 4 = extremely comprehensive/clear)	
Section	Content	Clarity
1. Introduction	3.3 (3, 3–4)	3.3 (4, 2–4)
2. Refining Research Question	4.0 (4, 4)	3.7 (4, 3–4)
3. Determining Study Design	3.3 (3, 3–4)	3.7 (4, 3–4)
4. Defining Implementation Outcome(s)	3.0 (3, 2–4)	4.0 (4, 4)
5. Selecting a Guiding Implementation Framework	3.7 (4, 3–4)	3.0 (3, 3)
6. Developing Data Collection Tools and Measures	4.0 (4, 4)	3.7 (4, 3–4)
7. Selecting Implementation Strategies	3.7 (4, 3–4)	3.3 (3, 3–4)
8. D&I Toolkit Resources	3.3 (4, 2–4)	3.3 (4, 2–4)

Based on the qualitative suggestions for improvement, we reordered the introduction section of the toolkit and reformatted all tables within the toolkit for consistency. We also added additional clarification in the implementation framework and implementation strategy sections to define terms and concepts more clearly. Lastly, after incorporation of external reviewer feedback, our internal telehealth team reviewed the toolkit again, revisions were finalized, we created a dissemination plan, a final draft was sent to the formatting team, the formatted version was reviewed by our team, and the toolkit was released as an interactive PDF to the HRSA Telehealth Center of Excellence Website.^32^

## Discussion

The expansion of telehealth in recent years has led to a proliferation of research demonstrating the potential for telehealth to increase health care access, improve patient outcomes, and lower costs.^33–35^ Moreover, patients and providers appreciate the convenience telehealth affords.^3,36^ These benefits, however, have neither been universal nor experienced equitably. Research has shown variation among practices in terms of their readiness and the resources available to them to implement telehealth. Similarly, despite telehealth being an effective tool to support health care access in rural communities, rural patients were less likely to engage in telehealth during the COVID-19 pandemic compared with urban patients.^9,37^ There also have been reported disparities in use of telehealth among different patient populations due to various demographic characteristics and social determinants of health.^8,9,11,34^

With the Centers for Medicare and Medicaid Services making permanent many of the telehealth flexibilities authorized during the pandemic and many state Medicaid and private payers following suit, it is evident telehealth will continue to play an important role in the US health care system and the evolving landscape of evidence-based interventions.^38,39^ D&I research has great promise to help stakeholders understand global processes and complexities related to telehealth clinical practice and support more equitable access to novel telehealth programs across care settings and among diverse populations.

Telehealth researchers and administrators have the opportunity to leverage D&I research to better understand the barriers and facilitators to telehealth implementation and sustainment and how to best adapt telehealth programs to meet their specific contexts. Despite the utility of D&I, many have noted barriers to engagement in D&I research due to lack of training and the diversity of frameworks, approaches, and tools within the field.^14^ Resources are needed to support uptake of D&I research within different scientific communities. Thus, a primary strength of this telehealth toolkit is that it meets a gap in the literature to provide D&I guidance specifically to the field of telehealth. The toolkit can be utilized in a variety of settings as a template that will inform development, implementation, and sustainment of telehealth innovations. The toolkit can also identify resource and logistical strategies that may be utilized to mitigate telehealth disparities among rural communities. With tools like this to support the development of D&I research questions and rigorous methods to evaluate telehealth outcomes, ultimately additional telehealth-specific D&I research can advance the development and implementation of appropriate and efficacious strategies to reach and provide quality care through telehealth.

Future directions for this telehealth toolkit include continued modification of the toolkit content and format by our interdisciplinary team to meet the dynamic D&I research field. Based on the external review, we can add introductory content to the toolkit in the future to make it more functional for a beginner D&I researcher. Our team is currently tracking ongoing dissemination of the toolkit by monitoring counts of toolkit downloads from the COE website and will plan future evaluations of usability and outcomes generated from toolkit use.

## Conclusion

With telehealth becoming a mainstay in the US health care system, it is vital to identify barriers and facilitators to implementation and sustainment of telehealth innovations. The field of D&I offers structured methods to assess implementation processes and outcomes. Developed by a multidisciplinary team of D&I scientists and telehealth experts, the toolkit described in this manuscript provides stepwise resources to guide telehealth teams and stakeholders in D&I evaluation.

## Authorship Contribution Statements

E.E.J.: Toolkit conceptualization, toolkit development and editing, manuscript composition and editing; R.K., R.V., E.W., and D.F.: Toolkit conceptualization, toolkit editing, and manuscript composition and editing; K.R.S.: Toolkit conceptualization, toolkit development and editing, and manuscript composition and editing.

## Abbreviations Used

AHRQ: Agency for Healthcare Research and Quality
COE: Telehealth Center of Excellence
D&I: dissemination and implementation

## References

[1] Khoshrounejad F, Hamednia M, Mehrjerd A, et al. Telehealth-based services during the COVID-19 pandemic: A systematic review of features and challenges. Front Public Health 2021;9:711762; doi: 10.3389/fpubh.2021.711762 34350154 PMC8326459

[2] Garfan S, Alamoodi AH, Zaidan BB, et al. Telehealth utilization during the Covid-19 pandemic: A systematic review. Comput Biol Med 2021;138:104878; doi: 10.1016/j.compbiomed.2021.104878 34592585 PMC8450049

[3] American Medical Association. Telehealth Survey Report; 2021. Available from: https://www.ama-assn.org/system/files/telehealth-survey-report.pdf

[4] Shigekawa E, Fix M, Corbett G, et al. The current state of telehealth evidence: A rapid review. Health Aff (Millwood) 2018;37(12):1975–1982; doi: 10.1377/hlthaff.2018.05132 30633674

[5] Bulkes NZ, Davis K, Kay B, et al. Comparing efficacy of telehealth to in-person mental health care in intensive-treatment-seeking adults. J Psychiatr Res 2022;145:347–352; doi: 10.1016/j.jpsychires.2021.11.003 34799124 PMC8595951

[6] Tsou C, Robinson S, Boyd J, et al. Effectiveness of telehealth in rural and remote emergency departments: Systematic review. J Med Internet Res 2021;23(11):e30632; doi: 10.2196/30632 34842537 PMC8665379

[7] Frasco MA, Trish E. Targeting Affordability in Healthcare: A Review of the Evidence. Schaeffer White Paper Series; University of Southern Califorina: Los Angeles, California; 2021.

[8] Eberly LA, Kallan MJ, Julien HM, et al. Patient characteristics associated with telemedicine access for primary and specialty ambulatory care during the COVID-19 pandemic. JAMA Netw Open 2020;3(12):e2031640; doi: 10.1001/jamanetworkopen.2020.31640 33372974 PMC7772717

[9] Pierce RP, Stevermer JJ. Disparities in the use of telehealth at the onset of the COVID-19 public health emergency. J Telemed Telecare 2023;29(1):3–9; doi: 10.1177/1357633x20963893 33081595 PMC7578842

[10] Uscher-Pines L, Arora N, Jones M, et al. Experiences of health centers in implementing telehealth visits for underserved patients during the COVID-19 pandemic: Results from the connected care accelerator initiative. Rand Health Q 2022;9(4):2. 10.7249/rhq-09-04-02 PMC9519102 36238021

[11] Patel SY, Mehrotra A, Huskamp HA, et al. Variation in telemedicine use and outpatient care during the COVID-19 pandemic in the United States. Health Aff (Millwood) 2021;40(2):349–358; doi: 10.1377/hlthaff.2020.01786 33523745 PMC7967498

[12] Bauer MS, Damschroder L, Hagedorn H, et al. An introduction to implementation science for the non-specialist. BMC Psychol 2015;3(1):32; doi: 10.1186/s40359-015-0089-9 26376626 PMC4573926

[13] Brownson RC, Colditz GA, Proctor EK. Dissemination and Implementation Research in Health: Translating Science to Practice. Oxford University Press: Oxford, United Kingdom; 2017.

[14] Stevens ER, Shelley D, Boden-Albala B. Barriers to engagement in implementation science research: A national survey. Transl Behav Med 2021;11(2):408–418; doi: 10.1093/tbm/ibz193 31958137

[15] Agency for Healthcare Research and Quality. Publishing and Communications Guidelines Section 6: Toolkit Guidance. Rockville, MD; 2021.

[16] Baumann AA, Morshed AB, Tabak RG, et al. Toolkits for dissemination and implementation research: Preliminary development. J Clin Transl Sci 2018;2(4):239–244; doi: 10.1017/cts.2018.316 30820360 PMC6382336

[17] Sterba KR, Johnson EE, Douglas E, et al. Implementation of a women's reproductive behavioral health telemedicine program: A qualitative study of barriers and facilitators in obstetric and pediatric clinics. BMC Pregnancy Childbirth 2023;23(1):167; doi: 10.1186/s12884-023-05463-2 36906564 PMC10007723

[18] Guille C, Johnson E, Douglas E, et al. A pilot study examining access to and satisfaction with maternal mental health and substance use disorder treatment via telemedicine. Telemed Rep 2022;3(1):24–29; doi: 10.1089/tmr.2021.0041 35720443 PMC8989094

[19] Kirkland EB, Johnson E, Bays C, et al. Diabetes remote monitoring program implementation: A mixed methods analysis of delivery strategies, barriers and facilitators. Telemed Rep 2023;4(1):30–43; doi: 10.1089/tmr.2022.0038 36950477 PMC10027345

[20] Johnson EE, MacGeorge C, King KL, et al. Facilitators and barriers to implementation of school-based telehealth asthma care: Program champion perspectives. Acad Pediatr 2021;21(7):1262–1272; doi: 10.1016/j.acap.2021.04.025 33940203

[21] MacGeorge CA, King K, Andrews AL, et al. School nurse perception of asthma care in school-based telehealth. J Asthma 2022;59(6):1248–1255; doi: 10.1080/02770903.2021.1904978 33730979

[22] MacGeorge C, Andrews A, Sterba K, et al. A mixed methods evaluation of the implementation of asthma care in rural school-based telehealth clinics. Pediatrics 2021;147:957–959; doi: 10.1542/peds.147.3MA10.957

[23] Johnson EE, MacGeorge C, Andrews A, et al. Nurse perspectives regarding implementation of an asthma monitoring mobile health application in the school setting. Telemed J E Health 2021;27(8):955–962; doi: 10.1089/tmj.2021.0100 34152858 PMC8432601

[24] Bian J, Cristaldi KK, Summer AP, et al. Association of a school-based, asthma-focused telehealth program with emergency department visits among children enrolled in South Carolina medicaid. JAMA Pediatr 2019;173(11):1041–1048; doi: 10.1001/jamapediatrics.2019.3073 31498379 PMC6735423

[25] Halterman JS, Fagnano M, Tajon RS, et al. Effect of the school-based telemedicine enhanced asthma management (SB-TEAM) program on asthma morbidity: A randomized clinical trial. JAMA Pediatr 2018;172(3):e174938; doi: 10.1001/jamapediatrics.2017.4938 29309483 PMC5885835

[26] Kim CH, Lieng MK, Rylee TL, et al. School-based telemedicine interventions for asthma: A systematic review. Acad Pediatr 2020;20(7):893–901; doi: 10.1016/j.acap.2020.05.008 32446856 PMC7241375

[27] Zhai YK, Zhu WJ, Cai YL, et al. Clinical- and cost-effectiveness of telemedicine in type 2 diabetes mellitus: A systematic review and meta-analysis. Medicine (Baltimore) 2014;93(28):e312; doi: 10.1097/md.0000000000000312 25526482 PMC4603080

[28] Luca DL, Margiotta C, Staatz C, et al. Financial toll of untreated perinatal mood and anxiety disorders among 2017 births in the United States. Am J Public Health 2020;110(6):888–896; doi: 10.2105/ajph.2020.305619 32298167 PMC7204436

[29] U.S. Department of Health and Human Services Substance Abuse and Mental Health Services Administration Center for Behavioral Health Statistics and Quality. National Survey of Drug Use and Health Releases. 2019.

[30] Forray A, Merry B, Lin H, et al. Perinatal substance use: A prospective evaluation of abstinence and relapse. Drug Alcohol Depend 2015;150:147–155; doi: 10.1016/j.drugalcdep.2015.02.027 25772437 PMC4387084

[31] Goldman-Mellor S, Margerison CE. Maternal drug-related death and suicide are leading causes of postpartum death in California. Am J Obstet Gynecol 2019;221(5):489.e1–489.e9; doi: 10.1016/j.ajog.2019.05.045 PMC6829056 31173749

[32] Johnson E, Sterba K. Implementation Science Telehealth Toolkit. MUSC Telehealth Center of Excellence: Charleston, SC; 2023. 10.1089/tmr.2023.0039 PMC10561742 37817872

[33] Totten AM, Womack DM, Griffin JC, et al. Telehealth-guided provider-to-provider communication to improve rural health: A systematic review. J Telemed Telecare 2022;1357633x221139892; In Press; doi: 10.1177/1357633x221139892 PMC11389081 36567431

[34] Hatef E, Wilson RF, Hannum SM, et al. Use of Telehealth During the COVID-19 Era. In: Agency for Healthcare Research and Quality (US): Rockville, MD; 2023. 37043587

[35] McCord C, Ullrich F, Merchant KAS, et al. Comparison of in-person vs. telebehavioral health outcomes from rural populations across America. BMC Psychiatry 2022;22(1):778; doi: 10.1186/s12888-022-04421-0 36496352 PMC9736702

[36] Berry CA, Kwok L, Massar R, et al. Patients' perspectives on the shift to telemedicine in primary and behavioral health care during the COVID-19 pandemic. J Gen Intern Med 2022;37(16):4248–4256; doi: 10.1007/s11606-022-07827-4 36167954 PMC9514672

[37] Pandit AA, Mahashabde RV, Brown CC, et al. Association between broadband capacity and telehealth utilization among Medicare fee-for-service beneficiaries during the COVID-19 pandemic. J Telemed Telecare 2023;1357633x231166026; In Press; doi: 10.1177/1357633x231166026 PMC10076155 37016902

[38] Health Resources & Service Administration. Telehealth Policy Changes After the COVID-19 Public Health Emergency; 2023. Available from: https://telehealth.hhs.gov/providers/telehealth-policy/policy-changes-after-the-covid-19-public-health-emergency [Last accessed: April 1, 2023].

[39] Center for Connected Health Policy. Spring 2023 Report: State Telehealth Laws & Medicaid Program Policies; 2023. Available from: https://www.cchpca.org/resources/state-telehealth-laws-and-reimbursement-policies-report-spring-2023/ [Last accessed: April 1, 2023].

